# Walking gait biomechanics in individuals with quadriceps tendon autograft anterior cruciate ligament reconstruction

**DOI:** 10.3389/fspor.2025.1546297

**Published:** 2025-05-09

**Authors:** Kate Pfile, Bennett Prosser, Harris Slone, Michelle McLeod, Chris Gregory, Jennifer Hunnicutt

**Affiliations:** ^1^ Department of Health and Human Performance, College of Charleston, Charleston, SC, United States; ^2^ Orthopedics and Physical Medicine & Rehabilitation, Medical University of South Carolina, Charleston, SC, United States; ^3^ Arthritis Foundation, Atlanta, GA, United States; ^4^ Health Sciences and Research, Medical University of South Carolina, Charleston, SC, United States; ^5^ Hunnicutt Writing and Consulting, LLC, Panama City, FL, United States

**Keywords:** kinematics, kinetics, vertical ground reaction force, anterior cruciate ligament reconstruction (ACLR), gait biomechanics

## Abstract

**Introduction:**

Walking is a vital movement, corresponding to physical activity, health, and independent living. Persistent abnormal lower extremity kinetics and kinematics during walking may influence long-term joint health. Anterior cruciate ligament (ACL) injuries are common sport-related knee joint injuries resulting in short- and long-term dysfunctional movement patterns. Re-establishing normal gait biomechanical patterns following ACL reconstruction (ACLR) is a universal long-term rehabilitative goal and indicator of restored function. The use of the quadriceps tendon (QT) graft technique by orthopedic surgeons is increasing and growing evidence suggests it's viable for ACLR. However, no information is available examining walking gait biomechanics in QT-ACLR patients. Our study evaluated three-dimensional hip and knee joint biomechanics during the stance phase of walking gait in patients with QT-ACLR by comparing the ACLR and nonsurgical limbs. We hypothesized hip and knee joint biomechanics will differ between the QT-ACLR and nonsurgical limbs during the stance phase of gait.

**Methods:**

We recruited a convenience sample of 14 patients with unilateral QT-ACLR ∼11 months post-surgery from an orthopedic surgery clinic. Three-dimensional hip and knee kinematics and kinetics and vertical ground reaction force were assessed while participants walked at self-selected speeds. Data were time-normalized from 0%–100% (% stance phase), and ACLR and nonsurgical limbs were compared using curve analyses with 95% confidence intervals. Cohen's d effect sizes identified clinical differences between limbs.

**Results:**

The ACLR limb was significantly different from the nonsurgical limb for knee flexion angle (1%–8% and 58%–85%), knee flexion moment (14%–23%), hip flexion moment (60%–67%), knee adduction angle (9%–32%, 92%–100%), knee adduction moment (53%–81%), hip frontal plane angle (0%–100%), hip abduction moment (31%–35% and 71%–76%), knee external rotation angle (0%–100%), knee internal rotation moment (55%–84%), hip transverse plane angle (20%–39% and 88%–100%), and hip internal rotation moment (56%–88%). All significant findings had large effect sizes (*d* > 0.8).

**Discussion:**

Three-dimensional biomechanical gait alterations are present at the knee and hip following QT-ACLR when comparing between limbs. This pattern is consistent with other ACLR graft types. Participants demonstrated gait patterns associated with quadriceps avoidance and reduced proximal forces during the loading response and terminal stance phases. Rehabilitation and functional movement programs should target these deficits.

## 1 Introduction

Injury to the anterior cruciate ligament (ACL) is one of the most common (1, 2) and costly (3, 4) sport-related knee joint injuries. ACL reconstruction (ACLR) attempts to restore stability and promote a return to normal kinematic and kinetic function. Autograft ACLR most commonly includes harvesting a graft from either the patellar, hamstring, or quadriceps tendons. Bone-patellar tendon-bone (PT) and hamstring tendon (HT) grafts are most popular. Use of PT autografts can lead to issues following surgery such as reduced instrumental laxity, anterior knee pain, and knee extensor strength deficits, while the HT graft can result in poorer patient reported outcome measures and knee flexor strength deficits (5, 6). The quadriceps tendon (QT) graft was introduced by Marshall et al. in 1979 (7) and has since gained traction with approximately 10% of orthopedic surgeons in 2020 reportedly using it for ACLR due to positive patient-reported outcomes, increased knee stability, and decreased risk of re-injury (8). A recent systematic review showed that anterior knee pain outcomes are similar between HT and QT grafts with higher pain scores for patellar tendon grafts. Further, QT grafts had similar functional outcomes and revision rates compared to PT and HT autografts (5).

Walking is one of the most essential human movements, corresponding to an active lifestyle, better overall health, and longer independent living (9, 10). Walking requires coordination across different systems and joints. There is consistent evidence that walking biomechanics differ following ACLR, specifically comparing between limb knee kinematics (11), kinetics (11–15), and vertical ground reaction force (vGRF) (16, 17). However, due to the relative novelty of the QT graft option, the literature is lacking a description of knee biomechanics following QT-ACLR during walking gait. As QT autografts become a more prevalent surgical option, information regarding repetitive, functional movements like walking gait are needed to determine if current rehabilitation and functional return considerations are germane following this surgery. Further, examining hip joint biomechanics may identify compensatory movement and loading patterns that contribute to lower extremity dysfunction (18). Abnormal gait, a deviation in temporal-spatial, kinematic, kinetic, or muscle activation patterns from an expected pattern (19), can add to the degeneration of articular cartilage especially in the knee joint, further reducing vGRF absorption and accelerating the onset of posttraumatic osteoarthritis (20, 21). It is reported that ten years following ACL injury approximately one-third of ACLR knees developed posttraumatic knee osteoarthritis, however no patients with QT-ACLR were examined (22).

Curve analysis is beneficial when evaluating gait as it allows the observation of the overall movement pattern rather than conducting analyses at discrete points such as the peak angle or force within a subphase of gait (23). Specifically, curve analysis can reveal differences at key subphases during stance such as loading response, midstance, terminal stance, and preswing. This allows practitioners to better recognize deficiencies and develop appropriate interventions (24). The purpose of our study was to evaluate three-dimensional hip and knee joint biomechanics during the stance phase of walking gait in patients with QT-ACLR by comparing the ACLR and nonsurgical limbs. We hypothesized that hip and knee joint biomechanics will differ between the QT-ACLR and nonsurgical limbs during the stance phase of gait.

## 2 Materials and methods

### 2.1 Study design

This investigation was part of a larger study examining functional outcomes of ACLR (31) that received IRB approval from Medical University of South Carolina and all participants provided informed consent to complete patient reported outcomes and a walking gait analysis in the biomechanics lab. Prior to their enrollment in the research study, all participants underwent primary ACLR with QT autografts performed by a single orthopedic surgeon. Grafts were harvested with a minimally invasive, all inside technique preventing the use of bone plugs. Suspensory fixation was performed on the femoral tibial side (25).

### 2.2 Participants

Participants were recruited from an orthopedic surgeon's clinic as a sample of convenience. Study staff reviewed the health records of patients with prior ACLR for the inclusion/exclusion criteria. Study staff called patients who met the following criteria to request their interest in participating in this research study. The inclusion criteria consisted of the following: 14–55 years of age, history of unilateral, isolated ACLR (with or without concomitant meniscus pathology) between six months to two years post reconstructive procedure using ipsilateral autografts harvested from the QT, and ACLR performed by a fellowship-trained orthopedic surgeon (SH). Exclusion criteria included history of lower extremity injury or surgery, including ACL retears and revisions within the past 6 months, multi-ligament reconstructions, an inability to walk without assistance from an orthotic, knee brace, or another person, and self-reported knee arthritis that would limit range of motion at the knee.

### 2.3 Procedures

Demographic measures including anthropometrics, the Tegner Activity Scale, time since surgery and presence of concomitant meniscus surgery and patient reported outcome measures including the Lysholm Score, International Knee Documentation Committee Subjective Knee Form (IKDC) and Knee Injury and Osteoarthritis Outcome Score (KOOS) were collected for each patient. A lower extremity biomechanical assessment was completed using an active marker set (PhaseSpace Motion Capture, Phase Space, Inc. San Leandro, CA). A single experienced Certified Athletic Trainer (JH) performed all testing, placing markers on the pelvis and lower extremities (ASIS, greater trochanters, medial and lateral epicondyles, medial and lateral malleoli, 1st and 5th metatarsals and distal 2nd toe) with marker clusters on the sacrum, anterior mid-femur, lateral mid-shanks, and dorsal mid-feet. Participants walked in their preferred walking shoes on a split-belt instrumented treadmill (Bertec, Columbus, OH) at a self-selected walking speed. Participants were given time prior to collecting the data to familiarize themselves with treadmill walking. Once familiar, data capture trials began where participants walked for at least 10 s to reach a steady state, followed by a 30 s period for data collection. Three successful 30 s trials were collected, and patients were given at least 30 s of rest between each trial.

### 2.4 Data processing

Three-dimensional kinematics were recorded at 120 Hz with a 16-camera motion capture system. Coordinates were interpolated over gaps smaller than 20 samples and resampled at 100 Hz. Bilateral GRFs were recorded at 2000 Hz. Data were then filtered using a 4th order Savitzky-Golay filter acting on 21 data points, resampled at 100 Hz and used to identify gait events during treadmill walking. The stance phase was defined as the period between initial contact and toe off, with thresholds defined as vGRF >20N and <20N, respectively. Kinetic and kinematic variables were normalized for time and reduced to 100 data points depicting 1%–100% of the stance phase of the gait cycle. Kinetic data were calculated using inverse dynamics of a six degree of freedom segment model. The six degrees of freedom refers to the three planes of motion (sagittal, frontal, and transverse planes) at both the hip and knee joints. Vertical ground reaction forces were normalized to body mass while joint moments were normalized by body mass and height and reported as external moments. Joint angles were reported in degrees. All data were processed and analyzed using custom LabVIEW (National Instruments, Austin, USA) programs.

### 2.5 Statistical analysis

A curve analysis was performed to identify between-limb differences across the stance phase of gait (23). The phases of the gait cycle were interpreted as 1%–16.6% loading response, 16.7%–50% midstance, 50.1%–83.2% terminal stance, 83.3%–100% preswing (26). For each kinetic and kinematic variable, the mean and 95% confidence interval (CI) was calculated and graphed for the nonsurgical and QT-ACLR limbs to identify between limb differences. No overlap in the between-limb confidence intervals for three consecutive percentages during the stance phase indicated a statistically significant difference (*P* < 0.05). All graphs were created using Microsoft Excel (Microsoft Corporation, Redmond, WA). Cohen's d effect sizes with pooled standard deviations were calculated using the average mean and standard deviation during the significantly different window for each statistically significant variable. Effect sizes were interpreted as small (*d* < 0.2), medium (*d* = 0.5), or large (*d* ≥ 0.8) (27).

## 3 Results

### 3.1 Demographics

The study participants included eleven males and three females, as shown in Table 1. The average time between the data collection period and surgery was 10.8 ± 5.8 months. The reconstructions were performed on six left knees and eight right knees, and all participants were right leg dominant. Averages of self-reported outcome measures are reported in Table 1.

**Table 1:** Demographic data.

Demographic	Participant (Avg ± SD)
Sex (M/F)	11M 3F
Age (years)	25.9 ± 9.8
Mass (kg)	83.2 ± 16.3
Height (cm)	177.0 ± 11.0
BMI (kg/m^2^)	25.0 ± 4.5
Months since surgery	10.8 ± 5.8
Tegner (post-ACLR at time of data collection)	6.6 ± 1.7
Self-selected walking speed (m/s)	0.82 ± 0.22
Lysholm	86.5 ± 7.5
KOOS- Sport	77.0 ± 15.6

### 3.2 Overview of main findings

There were significant differences in kinematics between the QT-ACLR limb and nonsurgical limb and each difference had a large effect size (Table 2). There were significant differences with large effect sizes in external joint moments between limbs for all three planes at both the hip and knee. There was no significant difference between limbs for vGRF.

**Table 2:** Differences in Hip and knee biomechanics between ACLR and nonsurgical limbs.

Biomechanical Variable	% Stance Phase	ACLR Limb Mean ± SD	Nonsurgical Limb Mean ± SD	Effect Size (95% CI)*
Kinematics (degrees)				
Knee flexion angle	1–8	6.6° ± 0.95	4.0° ± 1.4	2.2 (1.2, 3.1)
	58–85	6.6° ± 1.2	4.0° ± 1.5	1.9 (1.0, 2.8)
Knee adduction angle	9–32	6.4° ± 0.12	7.6° ± 0.25	−6.0 (−7.7, −4.2)
	58–81	4.6° ± 0.16	3.6° ± 0.27	4.7 (3.3, 6.2)
	92–100	6.5° ± 0.10	8.1° ± 0.70	−3.4 (−4.5, −2.2)
Knee transverse plane angle	0–100	14.2° ± 1.9	7.2° ± 2.0	−3.6 (−4.7, −2.4)
Hip frontal plane angle	0–100	1.1° ± 2.2	3.5° ± 2.0	1.2 (0.4, 2.0)
Hip ER angle	20–39	0.37° ± 1.1	2.0° ± 1.0	1.57 (0.7, 2.4)
Hip IR angle	88–100	5.1° ± 0.23	3.2° ± 0.13	−9.7 (−12.4, −7.1)
Kinetics (Nm/kg*m)				
Knee flexion moment	14–23	0.19 ± 0.08	0.28 ± 0.08	−1.2 (−1.9, −0.4)
Knee adduction moment	53–81	0.17 ± 0.01	0.23 ± 0.02	4.0 (2.7, 5.3)
Knee IR moment	55–84	0.06 ± 0.01	0.09 ± 0.01	2.1 (1.2, 3.0)
Hip flexion moment	60–67	0.26 ± 0.03	0.36 ± 0.03	−3.3 (−4.5, −2.2)
Hip adduction moment	31–35	0.71 ± 0.005	0.78 ± 0.005	13.5 (9.9, 17.0)
	71–76	0.62 ± 0.01	0.68 ± 0.01	6.3 (4.5, 8.0)
Hip ER moment	56–88	0.05 ± 0.01	0.08 ± 0.02	1.7 (0.8, 2.6)

Negative indicates a decreased moment or angle in the ACLR limb compared to the nonsurgical limb. Only effect sizes for statistically significant differences are reported. ER, external rotation; IR, Internal rotation.

#### 3.2.1 Sagittal plane

**Figure 1:**
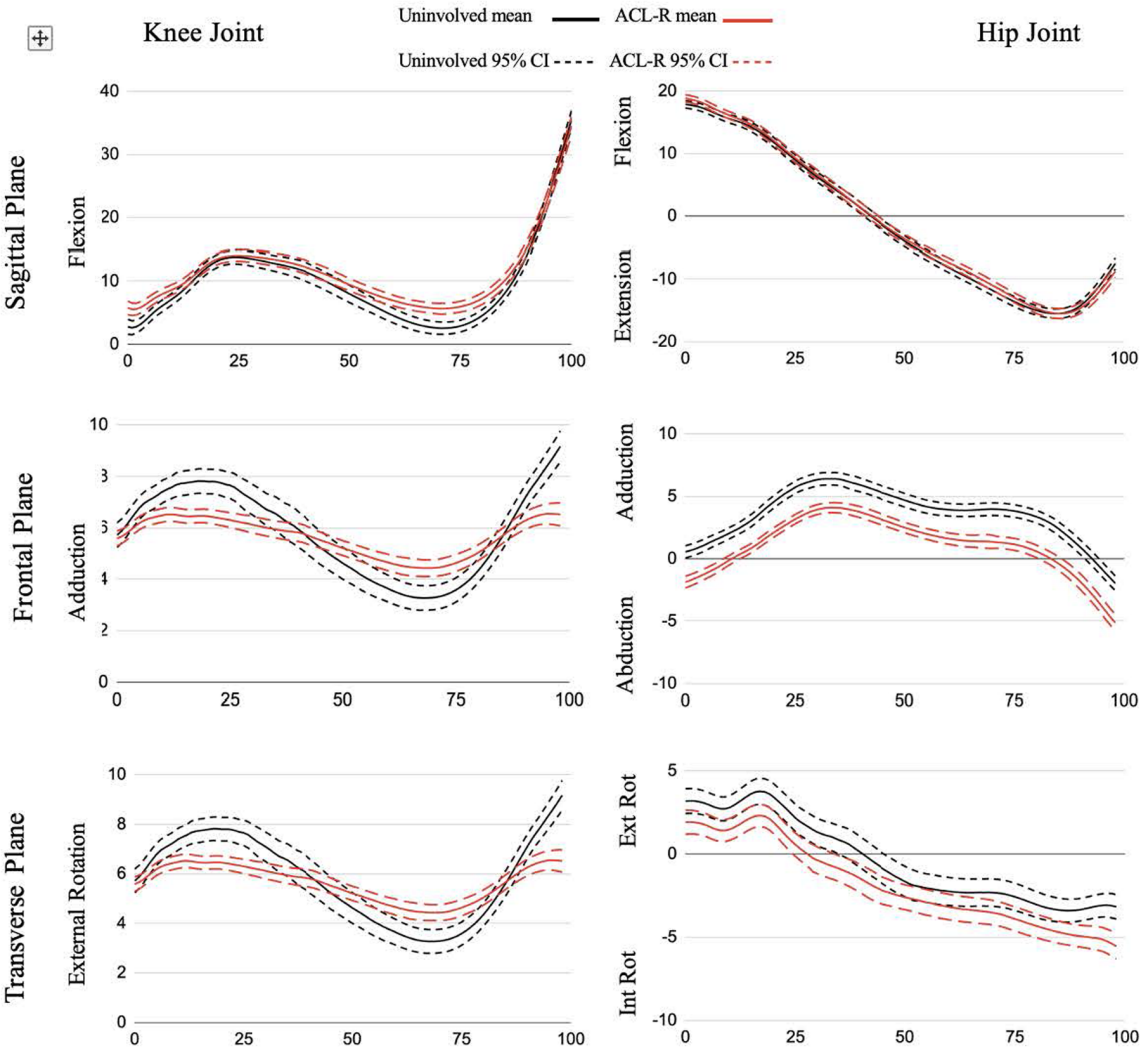
Joint angle ensemble curves for stance phase of gait. All data are presented in degrees on the y-axis across % stance phase on the x-axis.

At the knee (Figure 1), there was an increase in flexion angle in the ACLR limb compared to the nonsurgical limb from 1%–8% and 58%–85% of the stance phase as well as a smaller knee flexion moment from 14%–23% of stance. Additionally, the ACLR limb had a reduced hip flexion moment from 60%–67% compared to the nonsurgical limb.

#### 3.2.2 Frontal plane

**Figure 2:**
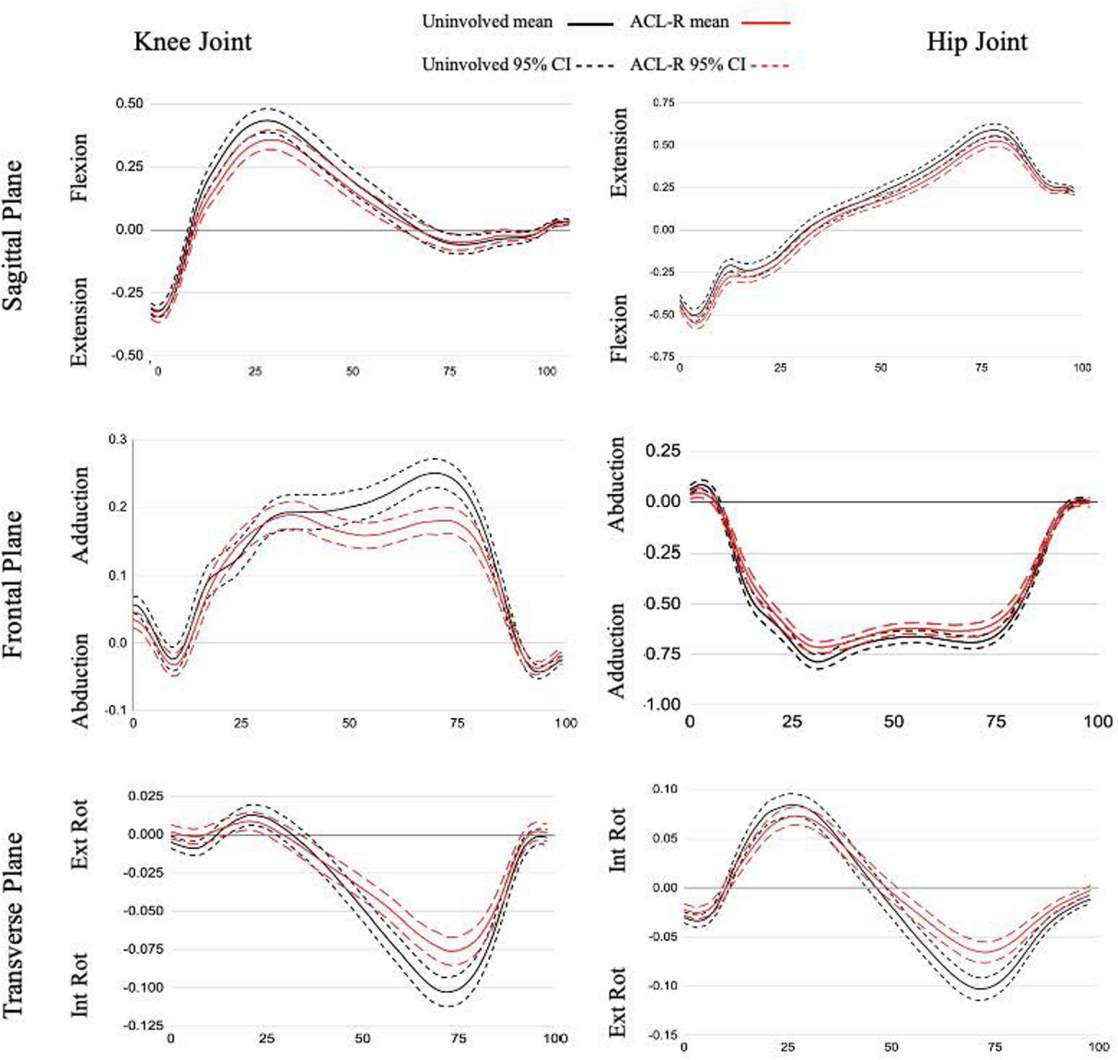
Joint moment ensemble curves for stance phase of gait. All data are presented as external moments (Nm/kg*m) on the y-axis across % stance phase on the x-axis.

The ACLR limb had a decreased knee adduction angle from 9%–32% and 92%–100% of the stance phase and an increased knee adduction angle from 58%–81% of the stance phase compared to the nonsurgical limb. There were decreases in knee adduction moment from 53%–81% compared to the nonsurgical limb. At the hip (Figure 2), in comparison to the nonsurgical limb, the ACLR limb was shifted toward more abduction and less adduction for the entirety of stance and showed a reduced hip abduction moment from 31%–35% and 74%–76%.

#### 3.2.3 Transverse plane

There was a reduction in knee external rotation angle for the ACLR limb throughout all of stance compared to the nonsurgical limb and a decreased knee internal rotation moment from 55%–84% in the ACLR limb compared to the nonsurgical limb (Figure 1). The ACLR limb also had a decreased hip external rotation angle from 20%–39% of stance phase and an increased hip internal rotation angle at 88%–100% of stance phase compared to the nonsurgical limb. The ACLR limb had a decreased hip internal rotation moment from 56%–88% compared to the nonsurgical limb (Figure 2).

## 4 Discussion

The purpose of our study was to evaluate three-dimensional hip and knee joint biomechanics during the stance phase of walking gait in patients with QT-ACLR by comparing the ACLR and nonsurgical limbs. Consistent with our hypothesis, the QT-ACLR limb displayed abnormal biomechanics in both kinematic and kinetic variables during stance compared to the nonsurgical limb; where most differences took place during the force absorption and propulsive periods of gait. However, unlike previous research on patients with ACLR, there were no significant differences between limbs for vGRF (16, 17, 28).

### 4.1 Loading response

Previous studies investigating walking biomechanics in other ACLR populations identified between-limb differences in both hip and knee joint kinematics (11, 28). A meta-analysis shows that stance phase peak knee flexion angle is significantly lower in the ACLR limb compared to the nonsurgical limb during gait (11). However, it is not feasible to determine exactly where within the stance phase the peak angle occurred due to the nature of the data extraction for this meta-analysis. Participants in our study displayed abnormal gait through significantly more knee flexion (terminal extension avoidance) compared to their nonsurgical limb during the loading response phase to allow for weight acceptance (Figure 1). Further examination revealed the participants' ACLR limbs displayed less knee flexion excursion (the difference between the maximum and minimum recorded knee flexion angles) with an average of 29° compared to 33° in the nonsurgical limb, exceeding the minimally clinically important difference of 3° (29). This “stiffened-knee strategy” where participants displayed less knee flexion excursion was also observed in patients with PT-ACLR (16) and ACL-deficiency (ACLD) (29). Participants may refrain from loading the joint in terminal extension due to on-going apprehension, a quadriceps strength deficit, or inadequate neuromuscular control (30). However, when compared to patients with PT-ACLR, those with QT-ACLR have demonstrated similar quadriceps cross-sectional area, isokinetic strength, and muscle activation, as well as functional hop test outcomes (31). Continued research of QT-ACLR functional outcomes beyond subjective questionnaires are needed in larger samples to better explain these findings. The literature shows that time since surgery can impact knee flexion angle in those who underwent ACLR (16, 28). Our study did not control for the specific time since surgery in which the gait analysis was conducted, thus it is possible that this variability (ranging from 6–23 months) may be a factor.

The ACLR limb had a decreased external knee flexion moment during the late loading response and early midstance phases when compared to the nonsurgical limb, as shown in Figure 1. This is the period when body weight is transferred to the lead stance leg and the head, arms, and trunk begin to align over the stance leg. Our finding is consistent with the existing gait literature examining peak knee flexion moment in patients with ACLR (11). Additionally, quadriceps weakness persists following ACLR (32) and previous research classifying ACLR patients (with a mixture of graft types) as weak or strong based on isometric quadriceps strength found those in the weak group displayed a reduced internal knee extension moment from 6%–72% of the stance phase (33). This suggests that diminished function of the quadriceps could also be the cause of reduced external knee flexion moment following QT-ACLR. In contrast, ACLR patients who completed criterion-based rehabilitation (including quadriceps strength symmetry >80%) showed no correlation between quadriceps strength asymmetry and internal knee extensor moment asymmetry (34). A recent meta-analysis (35) found quadriceps isokinetic strength limb symmetry was below 90% until 24 months post QT-ACLR. Further, there were no differences in strength between QT-ACLR and PT-ACLR populations serving as an explanation for why other studies including alternative graft types may also show an altered external knee flexion moment. Previously published research reported on this QT-ACLR patient sample identified asymmetry in quadriceps muscle strength and cross-sectional area outcomes, despite a high limb symmetry index for muscle activation (blinded citation). QT-ACLR rehabilitation should continue to emphasize both eccentric and concentric quadriceps strengthening along with gait training to promote quadriceps loading through terminal knee extension as a foundation to restoring function. Further research is needed to conclude whether quadriceps strength deficits cause aberrant knee biomechanics during walking and how these adaptations may affect long-term joint health.

### 4.2 Terminal stance

Participants in our study displayed significantly more knee flexion (terminal extension avoidance) compared to their nonsurgical limb during the loading response phase to allow for weight acceptance and the terminal stance phase to initiate propulsion (Figure 1). This latter phase difference is a similar finding to the early ACLR group (defined as 6 months-24 months post-ACLR) described by Goetschius et al. (28), and Gao et al. (36) who included a combination of ACLR auto- and allograft patients, and the weak quadriceps group in Pietrosimone et al. (33).

We also found decreased external knee adduction and internal rotation moments in the ACLR limb during the terminal stance phase. The reduced knee adduction moment occurs at the same time as the flattening of the knee adduction angle curve suggesting the kinematic pattern may be driving the kinetic pattern where a less adducted joint results in a lower joint torque when there is no difference in the external vGRF between limbs (37). Overall, the reduced joint moment peaks suggest an adaptive pattern to unload the joint and indicates a less dynamic strategy for frontal and transverse plane force management in the ACLR limb (Figure 2) (28). The ACL is loaded under an internal tibial rotation torque coupled with small (0–30) knee flexion angles (38). The significant transverse plane difference occurred between 55%–84% of the stance phase, corresponding to a gait phase with low knee flexion angles. Therefore, the reduction in transverse plane moment may be an effort to protect the ACL from high torques.

There are fewer studies reporting hip joint moments during gait in patients with ACLR. A meta-analysis synthesized 27 total studies (39), and only one (14) reported hip sagittal plane kinetics in comparison to a control group. Further, there were no results for frontal or transverse plane moments. More recently, Goetschius et al. (28) reported no differences in hip joint moments between ACLR and nonsurgical limbs. However, we found differences between limbs in all three planes during the terminal stance phase. Terminal stance is designated by the proximal leg advancing forward over the foot while the trunk also moves ahead of the support leg (26). Our results showed the QT-ACLR limb had reduced external hip extension and external rotation moments and a flattening of the hip adduction moment in both peaks during loading response and terminal stance. The reduced hip external rotation moment during the terminal stance phase may represent a neuromuscular adaptation to stabilize the proximal joint (40). As a whole, these kinetic differences may indicate a lack of proximal strength and/or neuromuscular control restoration during a relatively low demand functional task in patients 6–23 months post ACLR. A study comparing lunge biomechanics between pre- and post-ACLR time points in a small, mixed ACLR patient group (without QT patients) found a 15% increase in internal hip extensor moment. This points to a compensatory strategy with higher proximal force contribution following reconstruction that contrasts with our results (18). These are different patients, graft types, and tasks but both studies depict initial investigations into a complex question around lower extremity biomechanical patterns following ACLR. Gluteal strength is a common emphasis within ACLR rehabilitation programs, and a recent meta-analysis found no differences in hip strength outcomes between the ACLR limb and nonsurgical limb or healthy control (41), contrary to evidence of sustained thigh muscle weakness. Therefore, additional research is needed to better understand the driving force of the interlimb kinetic differences at the hip and influence on distal joint biomechanics and function. External hip joint moment magnitude is influenced by the position of the center of mass, so another future direction may be to examine trunk positioning during gait to further elucidate this finding (42).

### 4.3 Sagittal plane

There was no significant difference between limbs for the hip sagittal plane angle, which is consistent with the limited literature (28). However, without a control group we cannot specify whether the surgical limb returned to normal motion or if the nonsurgical limb adapted to maintain symmetry with the ACLR limb. Slater et al. (39) reported a peak mean hip flexion angle of 25° in the healthy control group and just under that for the ACLR group nonsurgical limb while our participants' peak flexion angles were 18° and 19° for the nonsurgical and ACLR limbs, respectively. Further, the meta-analysis shows that time post-surgery may influence this movement pattern because participants at 9 and 11 months post-ACLR had significantly more hip flexion with 37° and 33°, respectively (39). The average time since surgery for our participants fell within this time frame but they displayed only 50%–60% of the peak hip flexion. This discrepancy may be due to differences in graft type as this was not specified in the meta-analysis results. To our knowledge, this is the first study describing gait biomechanics for patients with QT-ACLR so this population would not have been included in the earlier meta-analysis. Grafting the central portion of the QT may influence function of the rectus femoris as a hip flexor and future investigation should examine hip flexor strength and neuromuscular control following QT-ACLR.

### 4.4 Frontal and transverse plane

A pattern of reduced excursion in the ACLR limb was present in the knee adduction angle (Figure 1). We found 2° of excursion in the ACLR limb compared to 6° in the nonsurgical limb. This represents less dynamic frontal plane motion, with less adduction during the loading response and preswing stance phases and more adduction during the midstance and terminal stance phases of gait. Fewer studies have reported frontal plane knee angle kinematics during walking and of those limited studies, the results are mixed (11) with some showing no difference between limbs (28), while others report a decreased peak knee adduction angle (14) or an increased peak adduction angle when compared to a healthy control group (43, 44). Participants may be limiting frontal plane range of motion due to changes in postural control post-ACLR (45) in an effort to stabilize the joint by limiting variability. Staying within a smaller envelope of motion throughout the stance phase of gait decreases their adaptability to various conditions and perturbations (46). The reduction in variability within our participants can be visualized (Figure 1) by the narrower confidence interval (driven by a smaller standard deviation) for the ACLR limb compared to the nonsurgical limb.

Research is inconclusive on frontal plane loading following ACLR. One study found an increase in peak internal knee abduction moment (corresponding to an increased external knee adduction moment) in patients with ACLR compared to a matched healthy control (43). Another showed no difference for peak external knee adduction moment when comparing PT-ACLR and HT-ACLR male patients however both groups displayed smaller peak moments when compared to a control group (14). While a third reported a decreased knee abduction moment compared to healthy matched controls (47). Frontal plane loading is significant as it influences the distribution of forces between the medial and lateral tibiofemoral joint compartments (14, 43) and corresponds to changes in cartilage composition measures (48). More specifically, in healthy cartilage a larger knee adduction moment improves medial cartilage thickness whereas in those with osteoarthritis a higher adduction moment is associated with reduced cartilage thickness (49). These differences in frontal plane biomechanics for the ACLR limb indicate abnormal biomechanics and the potential for changes to cartilage loading and homeostasis (49). Literature suggests that frontal plane loading strategies shift with time post-surgery where during the initial postsurgical phase (<2 years) external adduction moments are less than the nonsurgical limb but a reversed pattern with longer recovery time (>5 years) (28). Underloading, an adaptation to impairments such as pain, swelling, decreased range of motion, and muscle weakness immediately following ACLR, creates a “learned nonuse” phenomenon (50) and has been shown to be a contributor of poor cartilage health and potentially osteoarthritis (48).

The transverse plane knee angle showed a large and consistent effect between limbs where the ACLR limb had less tibial external rotation compared to the nonsurgical limb throughout the entirety of stance. A similar pattern was described in other ACLR (36) and ACLD patients (51). The difference in external rotation may stem from a loss of external rotation during the swing phase due to an insufficient screw-home mechanism. The screw-home mechanism occurs when the tibiofemoral joint approaches terminal extension and the tibia externally rotates approximately 15 degrees during the last 20 degrees of extension. From initial contact through the loading response phase the knee flexes and begins to reverse the screw-home mechanism moving towards internal rotation (52). While we did not report on swing phase kinematics, our participants displayed a more flexed knee at initial contact suggesting they may have never reached terminal extension and achieved the maximum amount of external rotation that couples with end-range extension. This gait pattern may be an unconscious strategy to limit anterior tibial shear force that occurs with open kinetic chain knee extension (38). Future research should include an analysis of the full gait cycle to better understand how kinematics during the non-weight bearing swing phase influence limb and joint positioning during the stance phase. Overall, these differences in tibiofemoral kinematics may alter contact patterns and affect load distribution to the meniscus and cartilage within the joint (16, 51). A longitudinal study found the ACLR cohort had changes in tibial rotation kinematics at six months that corresponded to cartilage matrix changes at the one year follow up (53). Therefore, over time, and millions of steps, altered kinematics may be a significant contributor to the development of posttraumatic osteoarthritis.

The curve analysis revealed a pattern at the hip of decreased adduction/increased abduction and decreased external rotation/increased internal rotation for the surgical limb. The QT-ACLR limb showed a significant and consistent ∼2.5° reduction in adduction throughout the entirety of stance. There was a ∼1–2.5° difference in transverse plane hip motion however this wasn't significant except for the preswing phase, where there was a trend toward more internal rotation (Figure 2). Evidence reporting frontal plane hip angle is limited (28, 39, 43) and absent for transverse plane within ACLR populations. The early ACLR group in Goetschius et al. (28) had more hip adduction during the swing phase but no inter-limb differences during stance. Our analysis did not include the swing phase, but future research should be conducted to better understand how the limb is positioned prior to loading. Another study (43) reported a peak hip adduction angle of 8.8° for patients with ACLR and 9.2° for healthy controls along with a hip adduction excursion of 9°. This peak is twice as high compared to our ACLR limb finding but in line with the total frontal plane excursion of 9°. The average time since surgery for the previously studied group was 5.4 ± 4.4 years, representing a wide range. Previous studies have shown different kinematic patterns over time when examining ACLR populations from a cross-sectional approach (28, 39) and a change in gait biomechanics between 6 and 12 months (16). Therefore, hip adduction angle may shift and “normalize” over time (28) but maintain a consistent motion excursion within the frontal plane.

### 4.5 Vertical ground reaction force

We hypothesized differences in vGRF between the ACLR and nonsurgical limbs; however, we did not find any statistically significant differences. The lack of a true control group hinders our ability to fully interpret this finding and determine whether force absorption patterns were restored in patients following QT-ACLR or if the symmetry is driven by adaptation within the nonsurgical limb. A study examining walking biomechanics in patients with PT-ACLR at 6- and 12-months post-surgery showed differences in vGRF patterns for both the ACLR and nonsurgical limbs compared to healthy controls suggesting that even the nonsurgical limb accommodated over time (16). There is a complex relationship between external loads and internal forces (from muscles) that may produce similar vGRF patterns between limbs, as we observed in our study, and potentially correspond to altered articular cartilage loading patterns.

A study investigating the effect of walking speed on vGRF symmetry in ACLR individuals found more interlimb symmetry at a slower walking speed compared to faster speeds. However, this finding was not apparent for healthy controls (54). Specifically, these participants determined their self-selected pace overground and then performed the walking trials on a split-belt instrumented treadmill. Our participants determined their self-selected pace on the split-belt instrumented treadmill and this likely influenced their walking speed as it is considerably slower (0.82 ± .22 m/s) than other reported studies with ACLR patients (16, 33, 54, 55).

### 4.6 Limitations

We note several limitations including a convenience sample of only 14 participants 6-months to 2-years post-surgery with an unequal distribution between males and females resulting in heterogeneity for both participant age and months since surgery, creating potentially more variability in the findings. However, all participants had returned to unrestricted physical activity and performed their gait analysis within this time frame, similar to the “early ACLR” group as defined in the Goetchius et al. (28) study allowing for comparison with their findings. Further, the case series study design did not include a control group limiting our ability to determine whether the nonsurgical limb compensated to maintain symmetry. Previous research appears to be mixed on whether there are biomechanical differences between the nonsurgical limb and a healthy control group, influenced by time since surgery (28). Emerging research points to limb dominance potentially influencing joint loading in ACL-R populations, but this was not accounted for in our analysis (56).

Participants had a slow average self-selected walking speed. Gait biomechanics are speed-dependent where lower walking speeds may result in reduced kinetics and joint ROM excursion. The lower self-selected walking speed for our participants may have affected the magnitude and symmetry of forces generated during the stance phase of the gait cycle and comparability across populations. We examined vGRF as a measure of limb loading but it does not fully represent joint loading that is also influenced by muscle forces and co-contraction that are not captured by external forces. Future study could include musculoskeletal modeling to better estimate joint contact forces. We didn't capture trunk kinematics which can influence distal joint moments (57) and this is an area for future researchers to examine as well as implementing statistical parametric mapping for a more comprehensive analysis of time-series data. However, the results remain valuable as they represent the function of a specific, novel research population.

### 4.7 Conclusion

This study is the first investigation into QT-ACLR walking biomechanics. Three-dimensional kinematic and kinetic gait alterations are present at the knee and hip in patients with QT-ACLR in the ACLR limb when compared to the nonsurgical limb, a pattern consistent with other ACLR patient graft types. Most of these differences occur in the periods of gait associated with higher forces, despite not finding significant differences in vGRF between limbs. In comparison to patient reported outcome measures, data describing gait biomechanics following QT-ACLR are extremely limited. Future studies should aim to test larger and more homogenous populations to better assess walking gait at specific recovery time points and examine multiple joints within the kinetic chain to evaluate for potential compensatory patterns. Researchers should work to compare gait biomechanics between patients with QT-ACLR and a true control group, knowing that nonsurgical limbs may also demonstrate biomechanical deficits following injury and ACLR. In line with ACLR rehabilitation protocols, the results of this study support emphasizing restoration of full knee extension, neuromuscular activation and strengthening of the quadriceps and proximal hip musculature to improve knee joint biomechanics and functional movement patterns.

## Data Availability

The raw data supporting the conclusions of this article will be made available by the authors, without undue reservation.
